# Humane Methods and Suggestions for Practice

**Published:** 1909-09-15

**Authors:** W. G. Ebersole


### HUMANE METHODS AND SUGGESTIONS FOR PRACTICE.

BY W. G. EBERSOLE, M.D.,D.D.S.

The first thing that a beginner in the practice of humanitarian dentistry must learn, is that he is to succeed he must commence at the foot of the ladder and climb up round by round.

The only advantage he has over those who are now meeting with success, is, that the ladder has been built, and all he has to do is to climb and receive the reward at the top.

Another thing that the beginner in the practice of this kind of dentistry must understand, is that it cannot be learned in a few minutes, hours or days. It requires much time and experience. The men who are its strongest advocates and who are the most successful in practicing it, are men who have devoted years to perfecting themselves in this direction.

In practicing humanitarian dentistry, the operator must deal with both real and imaginary pain. He must be as successful in handling one as the other, else he is not a success in this line of work.

Therefore, it is essential that we, in our discussion of this matter, take up both kinds of pain.

The first thing that presents itself to the dentist is the vivid imagination of the patients. This, coupled with the fear and dread most people have of dentists, and dental operations, places many of the patients in a condition bordering on irresponsibility. This condition must be reckoned with before attempting anything else.

To gain confidence, and dispel fear, must be the first thought of the dentist who desires to practice humanitarian dentistry.

Many men fail here. Most men make light of the patient's fear and lack of confidence, and a large majority of men have no patience with patients who imagine that they are being hurt. Success comes only when these patients are handled in a way which brings them to a position where they are capable of sound, rational judgment.

Let your patients know that you are fully aware of their condition and position in the matter, but impress upon them that they have your full sympathy, and that you are just as anxious not to hurt them as they are not to be hurt.

Tell them that in order not to hurt them, you are going to make use of methods and means, which make it possible to do their work free from pain, but that in doing this, you must have their hearty support. Caution them to notify you the moment they feel the slightest pain whatever.

*Make no promise to a patient which you do not expect to keep to the letter.*

*Cultivate a delicate touch. A touch can be cultivated which is both delicate and firm. A touch so light that it fails to irritate, and yet so full of strength and character that it inspires confidence.*

In preliminary examinations, if a cavity is found, that is enough. The knowledge that this cavity exists should suffice. Defer a complete examination as to the extent of that cavity, until you have made an appointment to care for that particular case and have plenty of time to investigate and treat existing conditions.

If a patient is suffering, or has been suffering, do not go probing until you have obtained the history of the case. Gain from information furnished an idea of what to expect, whether sensitive dentine, pulpitus, or peri-cementitis. Then proceed in the most cautious manner, to confirm this history, and, finding a condition, treat it, using every precaution not to hurt.

If the rubber is to be applied and there is the least likelihood of causing pain in so doing, have your assistant bathe the gums for a few moments with a solution of cocaine, adnephrin and water. If there is still danger of considerable difficulty in placing the rubber cloth, have the time lengthened, and the cocaine solution used until the gums show blanching.

Then proceed with the utmost care not to cause pain in adjusting the rubber. This is of the greatest importance in many cases. Many times have I seen patients in such a nervous state by the time the dam was in place that they were in no condition to be operated upon.

Having the rubber in place, dry away moisture, being careful not to cause pain in so doing.

Cautiously open up the cavity and remove all debris, using sharp chisels and excavators, resorting to the use of the engine and bur as little as possible, until you have ascertained the kind, nature and sensitiveness of the cavity.

Time and space will not permit discussion of the topical application of obtundents.

We should like to lay down a general rule, however, and that is, all topical remedies should be applied warm to sensitive cavities.

Humanitarian dentistry demands that all pain be eliminated, and a man is a success or failure just in proportion as he is able to do this. Producing pain at one time to avert it at another is not permissible.

If the cavity before us is one requiring more than topical remedies, we must next decide which of the remaining methods is best suited to the case in hand.

Following topical remedies, we consider high pressure anaesthesia next in importance. The high pressure instruments properly used make it possible to excavate the most sensitive teeth absolutely without pain.

Failure in the use of this method is not the fault of the instrument, unless the operator has abused it by careless manipulation.

The writer discovered a year and a half ago, a fact which he made public for the first time at Pittsburg, November 7, 1907, and that is that liquids can be forced through the enamel of the teeth with the high pressure syringe. It has formerly been taught that it was necessary to penetrate the entire layer of enamel before high pressure anaesthesia could be employed, but we have been able to demonstrate beyond any question that such is not the case.

It has been said that high pressure injures the pulp.

There is no more excuse for injuring or endangering the life of the pulp in the use of this method, than there is for the physician to injure or endanger the life of his patient in the administration of any of the many poisons that are used thousands of times daily in the treatment of the various diseases.

It is simply a question of dosage. The average dose is administered by the physician and continued, increasing or diminishing the dose, until the desired result is obtained. No two patients respond exactly alike, and in this, as in dentistry, each case is a case unto itself, and as such must be handled independently of all others.

At that moment when that part of the cavity farthest from the point of injection can be prepared painlessly, high pressure should cease if the best results are to be obtained.

Fear need not be created if the anaesthetic effect is extended considerably beyond this point.

The pulp may be anaesthetized with entire safety if necessary, but it is only in the most extreme cases that this is required. It is only when over-dosage or an improper solution is used that much danger exists.

Much injustice has been done both to humanitarian dentistry and to high pressure anesthesia by enthusiasts claiming this method suitable for all cases. It is but one means to an end. With it alone humanitarian dentistry must needs be a failure.

Not that every tooth in the head cannot be reached and thoroughly anaesthetized, but because in our intense desire to prevent pain, we lose sight at the time of equal importance.

There are many cases where I consider it decidedly inhumane to drill an extra cavity which cannot be included in the original one, when other methods at command grant equal immunity from pain without loss of extra tooth substance.

Cataphoresis? *Yes, I will dig up that supposed relic of the past. Bear with me while I do justice to a power which has fallen almost from sight because we, as a profession, fail to understand it, and would not give to it the necessary time to become familiar with the wonderful power for good which it holds and offers to the profession for the seeking thereof.*

Of the methods for obtunding sensitive dentine it offers most. There is no necessity of making extra cavities nor of bringing about general systemic conditions which will permit the cutting of sensitive dentine without the infliction of pain. Your dosage can be gauged accurately. There is no danger of carrying ptomaine poisoning into the pulp nor of creating depressing systemic conditions.

There are four drawbacks to its use, however.

First, the amount of time consumed in making the application.

Second, the difficulty in procuring suitable apparatus.

Third, the cost of perfect apparatus.

Fourth, the most difficult of all, is to teach the profession to handle it successfully.

There is nothing that the public will pay for so readily as the time spent in preventing pain.

A careful study of the case on hand permits the saving of much time by working on two parts of the mouth at the same time.

For instance, if there are a number of teeth that require filling, open up a cavity in one, insulate it by applying a second piece of rubber over the cavity and covering any metal filling with gutta percha, and then start cataphoresis on that case and while it is working, open up other cavities on the other side of the mouth.

Then when the first cavity is sufficiently obtunded, transfer your electrode to one of the newly opened cavities and complete your preparation of the first cavity and fill the same, and when through, the second cavity will be ready for thorough excavation.

Except for the time consumed in insulating and obtunding, this is an ideal method.

No man who attempts to practice humanitarian dentistry can afford to be without a cataphoric outfit, nor can he be without a high pressure syringe.

Men fail with these because they will not devote sufficient time to study that they may become expert in their use.

The busy man will not take time to, because he does not need to do so, while his patients will submit to the old way.

It is in the young men, therefore, that the possibility of humanitarian dentistry lies.

Before turning from local means for producing relief from pain and suffering, I wish to call your attention to the Leucodescent Therapeutic Lamp, with which I have been conducting experiments in a number of cases with very gratifying results.

This lamp is not a "cure all," but is a most valuable adjunct in the practice of all other therapeutic means. I do not know that it will be classed as a local agent, because its influence can be felt upon the entire system if properly applied. But I do know that in local affectations it can be used with decided benefit. I am not able to explain exactly how it produces its effect, but we do know that light is of universal necessity to the life and well-being of animal and vegetable cell development and sustenance. With this fairly understood, we are in a position to believe that the light rays may act much as do the sun rays in the production of life and health.

There is no question but what light applied with scientific intelligence as an adjunct to other means, will do more than any other one thing can do to restore the normal conditions physiologically.

I have been experimenting with the 500-candle power lamp, and the cases I have been handling have all had to do with dental lesions.

One case which is of greatest interest, is a lady about forty-six years of age who has been suffering for years with formation of pulp stones. She has been a sufferer to a greater or less extent from this affliction for a number of years in spite of all treatment given, and during a certain period in the month has always suffered agonies in her face and teeth.

Considering it a most unfavorable case for the Leucodescent treatment, and desiring to put the light at as severe a test as possible, I decided to see what could be accomplished by using it here.

Through the use of this method we have been enabled to give this patient two months of comfort and freeness from pain and suffering.

If when this patient was suffering intensely and we made use of the lamp, it was but a few moments until the pain subsided entirely, while the effect upon the nervous tissue was of the most soothing and quieting kind.

While seeking relief from pain only we were very much surprised to find after three or four treatments that the deafness of the patient which extended over a number of years, had been decidedly relieved, and after applying this treatment to the ears directly, we are able to say that at the present time the lady's hearing is as keen if not more acute than the majority of mankind.

Other cases in which we have used it with marked success, are for the pain following the extraction of abscessed teeth, and for the pain and soreness occasioned by the eruption of third molars, and in cases of odontalgia and pericementitis.

While of very decided value in the relief of pain and suffering, it is of inestimable value in extremely nervous patients. 'This is accomplished by using the blue rays which produce an anaesthetic or analgesic effect.

I have purposely avoided referring to a local anaesthesia producing by a hypodermic injection of cocaine or kindred preparations, because I believe that there are agencies at our command which make it possible to perform all necessary operations without resorting to the hypodermic needle. Of morphine, acetanelid, phenacitine and kindred preparations which act upon the nervous tissue in such a way as to render them partly inactive to stimulation, I cannot, in this paper, take the time to give them due consideration.

Of general anaesthetics, major and minor, I will say but little, because I believe that with the single exception of nitrous oxide gas, their place lies only in the surgical field, and yet, chloroform, ether, ethyl chloride and somnoform may be used in opening abscesses either through tooth or gum and in kindred operations. Chloroform is used by some men to produce the analgesic state when the excavation of sensitive dentine may be accomplished without pain to the patient.

Of general anaesthetics, nitrous oxide gas is the one par excellence for dental use.

Many substitutes have been offered and with each there have been announcements of great discoveries, but in time all have been compelled to admit that nitrous oxide gas stands in a class by itself as far as safety is concerned. As to the duration of the surgical stage, all depends upon how it is administered and for what purpose.

A number of men are using it with marked success in the preparation of sensitive cavities, placing the patient partially under the influence of the same.

Recently the writer has taken up the use of nitrous oxide and oxygen in the preparation of sensitive cavities by placing the patient in what is known as the analgesic state. We have not been working long enough with this manner of procedure to be able to state what the final outcome will be, nor to give you the result of this method of procedure as compared with the use of cataphoresis or high pressure anaesthesia.

But there are many places where this method can be used very advantageously, and where time is a question of importance, this method offers most, for with proper apparaatus, nitrous oxide and oxygen can be adjusted and administered so quickly that the time element may be reduced to the minimum.

We have been unable to make as free use of this method as the average practitioner might make, because of the fact that our patients have all been educated to have the pain element eliminated by other methods and means. And many of them do not take kindly to a change in this direction.

There are, however, many cases and conditions where it is a great time saver, and we say of this as we say of other means and methods described, that the human or humane dentist cannot well afford to be without an apparatus of this kind.

In all operations where freedom from pain cannot be obtained by the use of the means already cited, the writer maintains that the best interests of humanity demand the use of general anaesthetics even to the use of chloroform, if needs be.

Now in conclusion, let me say that the demand for anaesthetics or obtunding agents in dentistry is far greater than that of the medical profession. A physician may attend his professional duties day in and day out, and not be called upon to perform any act which would cause sufficient suffering to call for an anaesthetic. But with the dentist there is not a day that passes but that he will be required to make use of some agent to prevent his patient suffering torture, and therefore, it is the dental profession that should know most about anaesthetics and their use.

The failure to use more humane methods in performing dental operations, has caused a delay in the dental profession's obtaining proper recognition and standing.

The question has been asked: "How far are you justified by conditions presenting in dental practice in administering anaesthetics for the relief of the pain factor?"

In answer we would say that we are justified in using anaesthetics, either general or local, as the case may demand, until pain has been eliminated from all dental operations.—*The Journal.*

